# Heterogeneity of Characteristics among Housing Adaptation Clients in Sweden—Relationship to Participation and Self-Rated Health

**DOI:** 10.3390/ijerph13010091

**Published:** 2015-12-29

**Authors:** Björg Thordardottir, Carlos Chiatti, Lisa Ekstam, Agneta Malmgren Fänge

**Affiliations:** 1 Department of Health Sciences, Faculty of Medicine, Lund University, Box 157, Lund 221 00, Sweden; c.chiatti@inrca.it (C.C.); lisa.ekstam@med.lu.se (L.E.); agneta.malmgren_fange@med.lu.se (A.M.F.); 2 Italian National Research Center on Aging, Via S. Margherita 5, Ancona 60124, Italy

**Keywords:** activity limitations, cluster analysis, participation restrictions

## Abstract

The aim of the paper was to explore the heterogeneity among housing adaptation clients. Cluster analysis was performed using baseline data from applicants in three Swedish municipalities. The analysis identified six main groups: “adults at risk of disability”, “young old with disabilities”, “well-functioning older adults”, “frail older adults”, “frail older with moderate cognitive impairments” and “resilient oldest old”. The clusters differed significantly in terms of participation frequency and satisfaction in and outside the home as well as in terms of self-rated health. The identification of clusters in a heterogeneous sample served the purpose of finding groups with different characteristics, including participation and self-rated health which could be used to facilitate targeted home-based interventions. The findings indicate that housing adaptions should take person/environment/activity specific characteristics into consideration so that they may fully serve the purpose of facilitating independent living, as well as enhancing participation and health.

## 1. Introduction

People with disabilities living in their own homes are at risk for restricted participation in society, health problems and declining quality of life [1]. Since many societies have endorsed an aging-in-place policy, the number of people of all ages living in their own homes with disabilities will increase considerably over time [2]. This puts great demands on society to allocate appropriate resources toward effective home-based interventions in order to avoid participation restrictions and to promote health in the population.

Housing adaptations are common home-based interventions aiming at enhancing independent living in one’s own home and defined as the removal of physical environmental barriers and/or the readjustment of housing facilities [3]. The need for a housing adaption can arise due to an acute injury or disease, but more often this occurs after a period of functional decline. In many cases it is the home environment itself that is not designed to suit the change in level of ability during the life course of the individual. In Sweden, housing adaptions are tax-funded and the client is the formal applicant. The full costs of housing adaptions are borne by municipalities based on a need certification by a health professional. Accordingly, people applying for the grants have certified difficulties with independent living due to physical barriers in their homes [4,5]. They are at risk for declining activity, participation and health due to barriers in the physical environment and may need careful monitoring to avoid further decline.

Several studies in the field of housing adaptations have focused on the characteristics of the interventions [6,7,8,9] and related outcomes for the clients [5,10,11,12,13,14]. Such studies have provided valuable information about the processes and outcomes, however, the heterogeneity of the population receiving housing adaptations has not been considered *per se* as a focus of research. This lack of focus is particularly relevant as housing adaptations are clear examples of complex interventions, as outlined by the UK Medical Research Council [15], where the interaction of several different components contributed to the final outcome on clients. Pettersson *et al.* [16], for instance, showed that the process of housing adaptations is also affected by clients’ individual routines, habits, and expectations. So far, commonly available data regarding housing adaptation clients refer only to their age, gender and to the cost of the intervention borne by the municipalities [17]. In order to assure that resources allocated to interventions target the persons’ in most need of them, more knowledge about differences among people applying for housing adaptations is essential. Greater knowledge about their characteristics and heterogeneity can facilitate, for instance, the implementation of strategies for structured client baseline assessments and follow-ups [18,19,20]. This knowledge could serve as a preliminary step towards identifying clients most in need of interventions that facilitate their participation inside and outside the home and accordingly, allocate resources to those most in need.

Adapting the home environment can increase activity and participation, and have an important impact on the health among people with disabilities [5,10,11,12,21,22]. Important characteristics are often missing on official data. These are for example level of dependence in activities of daily living (ADL), a well-established measure of functional capacity [23] that research has demonstrated being strongly related to accessibility and usability in housing [5,24,25], health [26], and fall-related outcomes [27,28]. Research has also demonstrated relations between cognitive impairment, frailty and participation restrictions [29,30]. Generally, knowledge regarding these individuals’ characteristics is often lacking both at municipal and national level, hindering the possibility of assessing the overall impact of housing adaptation policies.

By deepening our understanding of housing adaptation clients’ characteristics and heterogeneity, it may be possible to distinguish those clients who require only a temporary or a single intervention from those for whom a stronger integration with other care professionals and a personalized, multicomponent approach is required. In addition, the ability to better differentiate between clients can be an effective method to improve the allocation of resources in times of economic constraints. From the perspective of the policy-makers, the identification of “clusters” of clients can support the implementation of new managerial instruments, as it happened in other care sectors. Today in Sweden, there are no official standards indicating how many resources a municipality should invest for specific groups of users, this leading to disparities in how users are treated in different areas across the country. In the health care sector of many countries instead, hospitals are reimbursed using the Diagnosis-related Groups system, a system which assigns the same reimbursement fees to each hospital for groups of patients with similar clinical conditions, thus assuring a more equal treatments among patients with similar needs [31]. A similar approach has been validated in the long-term care sector where the Resource Utilization Groups (RUG) system can be applied [32]. Last but not least, given the fact that participation and self-rated health are two overarching outcomes of health care and social interventions, knowledge about their distribution among housing adaptation clients would contribute to decisions on intervention at the client and societal levels, such as home health and social services.

This study is a first attempt to explore the complexity of housing adaptations clients which can serve as a preliminary step towards identifying the clients most in need of health care professional monitoring. The aim of this paper is to identify different groups of housing adaptation clients, and to investigate differences in participation frequency and satisfaction, as well as self-rated health among them.

## 2. Materials and Methods

### 2.1. Study Design

This study has a cross-sectional design and draws on baseline data from a larger longitudinal study of housing adaptation clients in three municipalities in southern Sweden [4].

### 2.2. Sampling and Participants

Individuals above the age of 20 were recruited by the occupational therapist responsible for the client in each municipality. Excluded were those living in sheltered housing and/or are unable to communicate or follow instructions in Swedish. The municipalities were chosen due to similarities in size and demographics. The participation rate was 42%. The major reason reported for not participating was ill health. In this study, baseline data on 178 individuals was included from the onset of the study.

### 2.3. Data Collection and Measurements

Data was collected using the applicant’s self-assessment and observations made during a home visit. The home visit took approximately 90 min. All data was collected by occupational therapists specially trained in the data collection methodology applied. Several aspects of housing and health were explored using validated instruments and study specific questions:

- *Dependence in ADL* was assessed with the ADL Staircase [33]. The instrument comprises nine items on feeding, transfer, using the toilet, dressing, bathing, cooking, transportation, shopping, and cleaning measured on a four point scale (0–3) as “independent without difficulty”, “independent with difficulty”, “partly dependent” and “dependent”, which are combined into a single-sum score (0–27) for dependence.
- *The number of functional limitations* was recorded using 12 items from the personal component in the Housing Enabler Instrument [34] with a higher score (0–12) indicating a larger number of functional limitations.
- *Cognitive impairment* was assessed with Montreal Cognitive Assessment (MoCA), in order to gather information on short-term memory, executive functions, visual-spatial abilities, language, attention, concentration, working memory, and temporal and spatial orientation. The scale is continuous (0–30) with “moderate impairment” (10–17 points), “mild impairment” (18–26 points) and “normal cognitive functioning” (more than 26 points) [35].
- *Concerns about falling* were measured using the short form of the Falls Efficacy Scale-International FES-I [36], which comprises seven activities: getting dressed or undressed, taking a bath/shower, getting in or out of a chair, going up or down stairs, reaching for something above head height or on the ground, walking up or down a slope and going out to a social event, assessed on a on a four point scale (1–4) with a higher score (7–28) indicating more concern. According to guidelines [36] the final FES-I score should be summarized using the person’s responses in 6 or 7 activities. In this study, we used an adjusted formula, taking those persons who responded only to 5 activities (e.g., owing to being unable to walk or using a wheelchair on a permanent basis) into consideration. The rationale for this choice was that the frailest group of the sample should not be excluded.
- *Satisfaction with usability in the home* was explored using the Usability In My Home (UIMH) Instrument [24], a self-rating instrument that measures client satisfaction with activity performance in relation to the design of the housing environment. Three components of usability were identified through an exploratory factor analysis; together these explained up to 65% of total variance: (a) the “self-care” component (5 items: going to the toilet, personal hygiene, preparing meals, preparing snacks and moving around the home with or without a mobility device); (b) the “social” component (3 items: socializing with family and friends in the home, contacting others via telephone or Skype, and watching TV or listening to the radio); (c) the “leisure and outdoor” component (3 items: entering the house, picking up the mail, engaging in hobbies and leisure activities in the home). A higher score (5–25 for self-care and 5–15 for the social and leisure-outdoor components) indicates more satisfaction with the usability of the home.
- *Self-rated health* was evaluated by means of the Euro-Qol 5D Visual Analogue Scale (EQ-VAS), a vertical scale ranging from 0 (worst imaginable health state) to 100 (best imaginable health state) [37].
- As a proxy measure of *participation*, six questions were developed to capture both frequency of, and satisfaction with, contacts in and outside the home, as well as the engagement in activities inside and outside the home, with others or alone. Three questions: (1) being in contact with friends, family or acquaintances at home; (2) doing something outside the home with friends, family or acquaintances; and (3) doing something outside the home alone were asked, each with two response scales, one for frequency and one for satisfaction The response scale for frequency was a five point scale: “almost never”, “yearly”, “monthly”, “weekly” or “daily”. Likewise, the response scale for satisfaction was: “very unsatisfied”, “unsatisfied”, “neither satisfied nor unsatisfied”, “satisfied” or “very satisfied”. For purposes of analysis, frequency of and satisfaction with participation were reclassified as follows: those participating “almost never”, “yearly” or “monthly” were classified as having “low frequency participation”, while those participating “weekly” or “daily” were classified as having “frequent participation”; similarly, those feeling “very unsatisfied”, “unsatisfied” or “neither satisfied or unsatisfied” were classified as being “unsatisfied” with participation while those feeling “satisfied” or “very satisfied” were classified as being “satisfied” with their participation.

### 2.4. Analytic Approach and Statistics

In order to investigate the characteristics and heterogeneity in the sample, cluster analysis was used to identify groups of housing adaptation applicants with similar characteristics. Cluster analysis is a set of exploratory statistical methods used in several disciplines to reduce the complexity of the information provided by a dataset. By using different types of algorithms, the analysis aims at creating clusters of individuals that are more similar to each other within the cluster than to those in other clusters. The aim of clustering methods is to explore and describe a phenomenon, rather than testing hypothesis.

Using SPSS Statistics 22.0 (IBM Corporation, Armonk, NY, USA) and STATA v.10.0 (StataCorp. LP, College Station, TX, USA), we applied a cluster analysis approach to our data in order to identify groups of housing adaptation applicants that were similar in terms of age, activity limitations, physical and cognitive impairments, fall-related concerns, and usability in the home. The variables included in the clustering where checked for correlations (Pearson’s *r* < 0.7) [38]. Hierarchical cluster analysis employing Ward’s method with squared Euclidean distances and standardized z-scores was chosen. Choosing the optimal number of cluster in a cluster analysis is often a difficult step. In our analysis, several solutions were tested (ranging from four to seven groups). After visualising the dendograms and considering the Calinski and Harabasz pseudo F-statistic [39], a six-group clustering solution was retained, as it better discriminated the groups according to all the variables of interests.

In a second step, the clusters identified by our analysis were used to further explore participation frequency and satisfaction and self-rated health. Chi-Square and Fisher’s exact test were used when comparing clusters on categorical data on participation and ANOVA were applied for self-rated health, with a *p* < 0.05 considered statistically significant.

### 2.5. Ethics

All subjects gave their informed consent for inclusion before they participated in the study. The study was conducted in accordance with the Declaration of Helsinki, and the protocol was approved by the regional Ethics Committee in Lund, Sweden (2012/566).

## 3. Results

Data on 124 individuals was available. Seventy-one % were women. For more details, see Table 1.

**Table 1:** Participant characteristics, *N* = 124.

Variable	Min-Max in the Sample	Mean (SD)
Age	36–95	75.2 (13.5)
Dependence in ADL	0–25	10.3 (5.5)
No. of functional limitations	0–9	3.7 (1.7)
Cognitive impairments	10–30	22.7 (4.8)
Concerns about falling	6–28	16.2 (5.6)
Usability In My Home:		
Self-care aspect–five items	6–25	19.0 (4.6)
Social aspects–three items	4–15	12.5 (2.8)
Leisure/outdoor aspect–three items	3–15	8.6 (3.7)

### 3.1. Heterogeneity Among Housing adaptation Applicants

We identified six clusters of housing adaptation applicants, comprising 12 (10% of the total sample) to 33 applicants (27% of the total sample). The clusters are characterized as follows:

- “*Adults at risk of disability*” (*cluster 1*; *n* = 15, mean age 49.7), characterized by low dependence in ADL, low number of functional limitations, no cognitive impairment and high level of concern about falling. Usability of their home was rated medium for self-care and leisure/outdoor aspects, high for social relations.
- “*Young old with disabilities*” (*cluster 2*; *n* = 23, mean age 70.7)*,* characterized by high dependence in ADL, high number of functional limitations, mild cognitive impairment, high level of concern about falling and all aspects of usability of their homes rated low.
- “*Well-functioning older adults*” *(cluster 3*; *n* = 20, mean age 78.8), characterized by low dependence in ADL and few functional limitations, mild cognitive impairments and low level of concern about falling. Usability for all aspects of housing was rated high.
- “*Frail older adults*” (*cluster 4*; *n* = 33, mean age 81.5), characterized by medium dependence in ADL and number of functional limitations, mild cognitive impairment and low level of concern about falling. Usability of their homes for all aspects was rated medium to high.
- “*Frail older adults with moderate cognitive impairments*” (*cluster 5*; *n* = 12, mean age 79.6), characterized by high dependence in ADL and high number of functional limitations, moderate cognitive impairment and low level of concern about falling. Usability of their homes was rated low for self-care and social aspects and medium/high for leisure/outdoor aspects.
- “*Resilient oldest old*” (*cluster 6*; *n* = 21, mean age 83.9)*,* characterized by low dependence in ADL, few functional limitations, mild cognitive impairment and a high level of concern about falling. Usability of their homes was rated high for self-care, medium for social aspects and low for leisure/outdoor aspects.

A detailed overview of cluster means is presented in Table 2.

**Table 2:** Characteristics of the six clusters identified (mean ± SD).

Characteristics	1. Adults at Risk of Disability	2. Young-Old with Disabilities	3. Well-Functioning Older Adults	4. Frail Older Adults	5. Frail Older-Moderate Cognitive Impairments	6. Resilient Oldest Old
Age	49.7 ± 8.0	70.7 ± 12.2	78.8 ± 6.9	81.5 ± 7.3	79.6 ± 8.0	83.9 ± 7.7
Dependence in ADL	8.0 ± 3.2	15.4 ± 4.6	4.1 ± 3.0	11.8 ± 5.1	13.8 ± 3.4	8.0 ± 3.3
Functional limitations	3.8 ± 1.3	5.2 ± 1.4	2.1 ± 1.1	4.1 ± 1.4	4.8 ± 1.3	2.6 ± 1.3
Cognitive impairments	27.0 ± 2.0	22.8±3.0	24.8 ± 3.4	21.0 ± 4.2	13.4 ± 2.9	25.5 ± 2.2
Concerns about falling	18.7 ± 6.0	22.1 ± 3.6	12.7 ± 3.2	12.7 ± 3.8	15.3 ± 6.4	17.5 ± 4.5
Usability in:						
-self-care	19.7 ± 4.3	14.4 ± 3.8	23.6 ± 1.3	19.8 ± 2.9	15.2 ± 4.3	20.3 ± 4.1
-social relations	13.8 ± 1.3	11.2 ± 2.4	14.7 ± 0.6	13.1 ± 2.0	8.1 ± 3.4	12.3 ± 2.7
-leisure/outdoors	8.9 ± 3.0	4.9 ± 2.6	10.4 ± 3.1	9.9 ± 3.5	10.0 ± 4.1	8.1 ± 3.0

### 3.2. Relationships between the Clusters, Participation and Self-Rated Health

Participation at home was reported as frequent for all the subjects in the “Well-functioning older adults” (cluster 3) and “Frail older with moderate cognitive impairment” (cluster 5) clusters. Ninety percent of the “Well-functioning older adults” (cluster 3) and 88% of “Frail older adults” (cluster 4) were satisfied with their participation at home. Least frequent and satisfactory participation at home was reported by “Young old with disabilities” (cluster 2). Differences in participation frequency and satisfaction at home were significant between clusters (*p* < 0.05).

Participation outside the home with others was reported as frequent by 65% and satisfactory by 80% among the “Well-functioning older adults” (cluster 3). Least frequent participation outside the home with others was reported in “Frail older with moderate cognitive impairment” (cluster 5) or 8% of the cluster. Least satisfaction with participation outside the home with others (39%) was found in “Young old with disabilities” (cluster 2). Differences in participation frequency outside the home with others were significant between clusters (*p* < 0.05).

Participation outside the home alone was reported as frequent and satisfactory by 70% among “Well-functioning older adults” (cluster 3). The lowest frequency (26%) and satisfaction (22%) were reported among “Young old with disabilities” (cluster 2). No significant differences between clusters were found. Self-rated health was lowest among “Young old with disabilities” (cluster 2) and highest among “Well-functioning older adults” (cluster 3), with significant differences between the clusters (*p* = 0.001). An overview of the number of individuals (and percentages) in each cluster is presented in Table 3.

**Table 3:** Relationship between clusters and participation and self-rated health.

Participation and Self-Rated Health	1. Adults at Risk of Disability	2. Young Old with Disability	3. Well-Functioning Older Adults	4. Frail Older Adults	5.Frail Older with Moderate CI ^a^	6. Resilient Oldest Old	Total Sample *N* = 124	*p*
Participation no. (%)								
-Frequently at home	11 (73)	16 (70)	20 (100)	29 (88)	12 (100)	18 (86)	106 (85)	0.025
-Satisfactory at home	10 (67)	12 (52)	18 (90)	29 (88)	8 (67)	17 (81)	94 (76)	0.036
-Frequently out w/others	8 (53)	6 (26)	13 (65)	13 (40)	1 (8)	10 (48)	51 (41)	0.017
-Satisfactory out w/others	7 (47)	9 (39)	16 (80)	22 (67)	6 (50)	14 (67)	74 (60)	0.061
-Frequently out alone	8 (53)	6 (26)	14 (70)	15 (45)	4 (33)	9 (43)	56 (45)	0.092
-Satisfactory out alone	8 (53)	5 (22)	14 (70)	15 (45)	4 (33)	9 (43)	55 (44)	0.059
Self-rated health (mean)	52.3	43.9	72.1	56.3	59.7	57.0	56.5 ^b^	0.001

^a^ CI = Cognitive Impairment; ^b^ *N* = 121.

## 4. Discussion

The results of our study add valuable knowledge about the heterogeneity among housing adaptation applicants in terms of activity, physical and cognitive functioning, as well as the usability of the environment. Our results also demonstrated differences in participation inside and outside the home, with others or alone, as well as differences in self-rated health between groups highlighting the need for targeted interventions to address the needs of these specific groups. To the best of our knowledge this is the first attempt to explore the heterogeneity among housing adaptation applicants, and while the results should be considered preliminary there are findings of interest to discuss.

Given the age distribution among housing adaptation clients it seemed obvious that the youngest applicants emerged as a cluster of its own (cluster 1). In spite of the fact that they were rather independent in ADL and had few physical functional limitations, they were very concerned about falling. One explanation could be that our definition of dependence related to dependence on other persons in daily activities. There might not be another person available during major parts of the day, forcing them to manage on their own even if they were afraid of falling. In comparison, the participants in cluster 2, who also had high concerns about falling, were more dependent and had more functional limitations than cluster 1, and they also had mild cognitive impairments. Concerns about falling emerge as a relevant issue for housing adaptation as fear of falling has been associated with long-term risk of disability [40], pervasive activity limitations and participation restrictions [41,42,43]. The findings indicate that for applicants with high concerns about falling when performing daily activities there is need for regular follow-ups that focus explicitly on these concerns, preferably integrated with other interventions that concern their everyday life at home.

It was also expected that “Well-functioning older adults” would gather into one cluster (cluster 3). This seems to be a group of housing adaptation applicants that, in spite of their need for having environmental barriers removed, seems to be quite independent and well-functioning. Given the well-known positive relationship between housing and functioning ability in the aging population [27], the effectiveness of housing adaptations (e.g., in terms of prevention of further functional decline) might even be stronger in this group of clients.

Of specific concern for all home-based health care is the increasing number of people with cognitive impairments living in their own homes. Also, the risk of cognitive decline increases with age [44]. Not surprisingly, in light of their higher mean age, clusters 4, 5 and 6 had more cognitive impairment than clusters 1, 2 and 3. Earlier, research has established a relationship between cognitive function and fear of falling [45,46]. However, similar to the findings of Uemura *et al.* [46], cluster 5 raises specific concerns since it was the only cluster where the included applicants had moderate cognitive impairment as demonstrated by the lowest mean score on MoCA, but were not among those most concerned about falling. This is probably due to their dependency on other people, *i.e.*, they are more likely to have more support in ADL than for example cluster 1.

Declines in health and older age often lead to changes in which participation gradually recedes from activities outside the home to participation in the home [47]. We found broad differences among clusters under the three aspects investigated. Low reports of participation both in and outside the home, as well as low self-rated health among “Young old with disabilities” and “Adults at risk of disability” (cluster 1 and 2) indicate the need for investigating participation restrictions in and outside the home beyond the environmental barriers. This is especially interesting since older applicants (e.g., cluster 3) seemed less affected in their participation. One explanation might be that being restricted to the home can be perceived as a natural consequence of ageing [47], while younger people may have higher expectations of participation with peers and therefore likely to feel dissatisfied if restricted to the home. For the younger applicants in clusters 1 and 2, concerns about falling may also be an important component of their participation restrictions. Concerns about falling are known to restrict activities and participation among community-dwelling older adults [13,41,42,48], while they are less explored among young adults [43]. The substantial difference in ADL dependence, functional limitations and concerns about falling between clusters 1 and 2, supports that it is not only dependence in ADL, but possibly the combination of dependence and concerns about falling that seem to restrict participation. In this context, it seems that other person/environment/activity specific characteristics rather than dependence in ADL should be taken into consideration when assessing and evaluation housing adaptations, so that they may fully serve the purpose of facilitating independent living, as well as enhancing participation.

While reports of participation outside the home and self-rated health are low for both “Frail older adults” (cluster 4), and “Resilient oldest old” (cluster 6), their reports of satisfaction with participation outside the home with others are higher and may indicate their need and use of support outside the home. Social support is a well-known indicator of participation [49,50] and health [51]. In light of this, the reports of low participation frequency and satisfaction outside the home among “frail older with moderate cognitive impairments” (cluster 5) raise specific concerns. Our findings support that people with cognitive impairments very often do not receive enough help to be active and participate in society [52]. The low self-rated health among “Frail older with moderate cognitive impairment” was to be expected, since cognitive function [29] and disability [53] are related to frailty; all in all, this group of people seem to be in need of more support than they receive. Support can be both formal and informal, however, it is important to know more about the nature and extent of support needed to facilitate participation outside the home, as engaging in social activities is especially beneficial for older adults in reducing disability, enhancing cognitive health, and higher self-rated health [54,55,56].

Other studies confirm the variability we found in the health and functional profiles of older adults [52,57,58,59]. The challenge faced by those providing a housing adaptation is therefore complex. Systematic and evidence based assessment of the needs among applicants and how their needs change over time is a prerequisite for effective follow-ups. Initiatives towards careful and systematic housing adaptation process, have been proposed [7,60] and the clusters identified in this study could be useful in this respect. Moreover, the financial burden posed by the increasing need for care of the aging populations will continue to contribute significantly to dramatic increases in health-care spending [61,62], stressing the need for targeted interventions that facilitate participation and health.

A methodological strength of this study is the rigor of its data collection procedure. Considerable efforts were put into training data collectors and providing continued methodological support, to increase the validity and reliability of the data. However, our sample did not include many people with severe cognitive impairments since they were unable to participate in the assessments due to e.g., disability, low mood or lack of stamina, which also generated internal dropouts for cognitive impairment data specifically. Probably, the prevalence of cognitive impairments is higher among Swedish housing adaptation clients. As a consequence of this limitation, it is likely that the relative size of cluster 5 (frail older people with moderate cognitive impairment) may be higher in the real life population than what is indicated in our results. Other concerns about data also relate to concerns about falling as measured using the FES-I short version instrument [36]. Participants using wheelchairs most often were unable to respond to the question “Are you afraid of falling when using the stairs?”, since they never used them. According to the manual the questions should be responded to hypothetically, but this seemed difficult to some participants.

While this study provided empirical support for heterogeneity among the applicants studied, it is limited in generalizability due to the participation rate and internal missing data. Previous studies of housing adaptations have employed a variety of instruments, making them difficult to compare [27]. Nonetheless, our sample does not differ considerably from other studies as what concerns age and gender distribution [5,11]. The clusters proposed in the paper should be further explored using data from larger samples of housing adaptation clients in order to assess their further generalizability. Moreover, the cross-sectional design does not capture changes in the sample over time. Further exploration of housing adaptation clients using longitudinal datasets could also be informative in assessing how clients' characteristics and needs tend to change over time.

## 5. Conclusions

Our study represents the first attempt to identify homogenous clusters in a heterogeneous sample of people applying for housing adaptations. This knowledge can serve as a preliminary step towards identifying clients most in need of interventions that facilitate their participation inside and outside the home and accordingly, allocate resources to those most in need.

The findings provide useful knowledge to health care professions for tailoring not only housing adaptations but also other complex interventions in peoples’ homes, aiming at enhancing participation and health.
